# Endoscopic versus microscopic transsphenoidal pituitary adenoma surgery: a meta-analysis

**DOI:** 10.1186/1477-7819-12-94

**Published:** 2014-04-11

**Authors:** Yang Gao, Chunlong Zhong, Yu Wang, Siyi Xu, Yang Guo, Chenyang Dai, Yan Zheng, Yong Wang, Qizhong Luo, Jiyao Jiang

**Affiliations:** 1 Department of Neurosurgery, Renji Hospital, School of Medicine, Shanghai Jiao Tong University, Shanghai 200127, China; 2 Department of Obstetrics & Gynecology, Renji Hospital, School of Medicine, Shanghai Jiao Tong University, Shanghai 200127, China

**Keywords:** Meta-analysis, Pituitary adenoma, Endoscopic, Transsphenoidal, Microscopic

## Abstract

**Background:**

Endoscopic transsphenoidal surgery has gradually come to be regarded as a preferred option in the treatment of pituitary adenomas because of its advantages of improved visualization and its minimal invasiveness. The aim of this study was to compare and evaluate the outcomes and complications of endoscopic and microscopic transsphenoidal surgery in the treatment of pituitary adenomas.

**Methods:**

We performed a systematic literature search of MEDLINE, EMBASE, the Cochrane Library and the Web of Science between January 1992 and May 2013. Studies with consecutive patients that explicitly and fully compared endoscopic and microscopic approaches in the treatment of pituitary adenomas were included.

**Results:**

A total of 15 studies (n = 1,014 patients) met the inclusion criteria among 487 studies that involved endoscopic surgery and 527 studies that dealt with microscopic surgery. The rate of gross tumor removal was higher in the endoscopic group than in the microscopic group. The post-operative rates of septal perforation were less frequent in patients who underwent endoscopic surgery. There was no significant difference between the two techniques in the incidence rates of meningitis, diabetes insipidus, cerebrospinal fluid leak, epistaxis or hypopituitarism. The post-operative hospital stay was significantly shorter for the endoscopic surgery group compared with the microscopic surgery group (*P* < 0.05). There was no significant difference in the length of the operation (*P* > 0.05).

**Conclusions:**

The present study indicates that the endoscopic transsphenoidal approach is safer and more effective than microscopic surgery in the treatment of pituitary adenomas.

## Background

In the late nineteenth century, the resection of a pituitary tumor via an open craniotomy was first described by Horsley [1]. Since then, the field of pituitary surgery has undergone constant evolution. Schloffer *et al*. [2] were the first to report the transsphenoidal approach in a sella tumor in 1907. It was Cushing *et al*. [3] who abandoned external incisions and popularized the sublabial transseptal transsphenoidal technique. In the 1960s, Hardy [4] perfected Cushing’s approach with the introduction of the operative microscope. The traditional transseptal/translabial approach has long been considered as the standard approach because it is associated with minimal morbidity and mortality. In recent years, with the development of endoscopic instruments and techniques, Jankowski [5] proposed a fully endoscopic approach to pituitary surgery in 1992. Currently, endoscopic transsphenoidal pituitary surgery has become a preferred alternative option because of its advantages of improved visualization and minimal invasiveness, which allows surgeons to gain access to central skull base lesions. However, the endoscope has the disadvantage of lacking the stereoscopic view obtainable with a microscope, which makes the benefits of the two techniques equivocal when comparing them in the treatment of pituitary adenomas.

The purpose of our study was to evaluate the outcomes and the complications associated with these two techniques by comparing endoscopic with microscopic surgery in the treatment of pituitary adenomas through a meta-analysis of the current relevant literature.

## Methods

### Search strategy

We performed a systematic search of MEDLINE, EMBASE, the Cochrane Library and the Web of Science for relevant literature between January 1992 and May 2013. We identified all relevant published and unpublished primary studies via an exhaustive search strategy. The following search terms were used: ‘pituitary’, ‘pituitary and surgery’, ‘endoscopic and pituitary’, ‘endoscopic/endoscopy’, ‘microscopic/microsurgery’, ‘transsphenoidal and surgery’. We browsed the abstracts and titles of primary collections and extracted all observational studies. Potentially relevant articles were considered by double evaluation. Additionally, the references of all obtained studies were reviewed for possible inclusion. The results were searched for humans and the English language.

### Inclusion/exclusion criteria

Studies were deemed appropriate for inclusion if they met the following criteria: 1) a direct comparison between fully endoscopic and microscopic (sublabial, transeptal) approaches for pituitary adenoma; 2) retrospective studies that included consecutive patients; and 3) each compared group included 10 or more patients who had undergone surgery in the same center. The exclusion criteria were as follows: 1) endoscopic-assisted comparison studies, single-armed studies or non-human studies; and 2) non-investigative studies (technical reports, case series, letters, and comments).

The search results were assessed independently by two authors (Siyi Xu and Yang Guo). Any disagreement was resolved unanimously by discussion.

### Methodological quality

The quality assessment of the retrospective comparative study was performed based on the Newcastle-Ottawa Scale [6], a scale that is also recommended by the Cochrane Non-Randomized Studies Methods Working Group. Each study was graded as ‘I’ if the score was >6 or as ‘II’ if the score was ≦5.

### Statistical analysis

Review Manager, version 4.2 (Revman, The Cochrane Collaboration; Oxford, UK) was used for the meta-analysis. All outcomes considered in this study were dichotomous and, therefore, proportions with their corresponding 95% confidence interval (CI) are reported. We chose to use the odds ratio (OR) as the summary statistic (an OR >1 favors the endoscopic group for gross tumor removal (GTR), whereas an OR <1 favors the endoscopic group for observed complications). Tests for heterogeneity were performed with the Chi-square and I^2^ for each meta-analysis. A fixed effects model was used when no heterogeneity (*P* > 0.05, I^2^ = 0%) or minimal heterogeneity (*P* > 0.05, I^2^ < 25%) was present, while a random effects model was applied in the presence of high heterogeneity (*P* < 0.05, I^2^ > 50%). The length of the operation and of the hospital stay were analyzed by using the equal-variance *t*-test (SPASS 19.0) and were considered significant if the *P* value was <0.05. In addition, the effect of publication and selection bias on the summary estimates was tested by both the Harbord-Egger bias indicator and Begg-Mazumdar bias indicator. A two-tailed *P*-value <0.05 was considered statistically significant.

### Analyzed items

The primary data items for this meta-analysis were: 1) the GTR, based on either post-operative imaging and/or normalization of hormonal hypersecretion that confirmed the absence of any tumor; 2) the length of the hospital stay and operative time of each operation; and 3) complications (post-operative cerebrospinal fluid (CSF) leak, diabetes insipidus (DI), hypopituitarism, meningitis, epistaxis, septal perforation).

## Results

**Figure 1:**
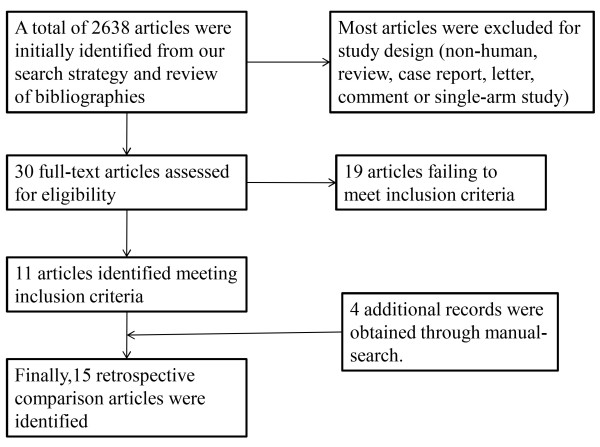
Flowchart diagram of the study selection process.

A total of 2,638 articles were initially identified using our search strategy and review of bibliographies. These articles were examined to exclude irrelevant studies, resulting in 30 potentially eligible articles. Subsequently, the full texts of these studies were examined thoroughly, and 19 articles were excluded based on their failure to meet the inclusion criteria. Four additional records which meet the inclusion criteria were obtained through a manual search. Ultimately, 15 articles retrospectively comparing endoscopic versus microscopic surgery in the treatment of pituitary adenomas were identified (Figure 1). The characteristics of the included studies are summarized in Table 1 and Table 2. All of the included reports were retrospective studies published between 1992 and 2013. A total of 1,014 patients was reviewed (endoscopic group = 487, microscopic group = 527). Unfortunately, large, prospective, randomized studies comparing the two techniques were not available because of the lack of relevant reports. According to the selected criteria of methodological quality, eleven studies [7-17] were identified as grade ‘I’ and four studies [18-21] were identified as grade ‘II’. To identify potential sources of the observed heterogeneity and to test the stability of our results, a sensitivity analysis was further performed by removing the grade ‘II’ studies.

**Table 1:** Characteristics of publication year, study type, cases in each group and GTR, length of operation and hospital stay for included studies

**Study**	**Publication year**	**Study type**	**Cases in each group**		**Cases of GTR**		**Length of operation (mean, min)**		**Length of hospital stay (day)**	
			**E**	**M**	**E**	**M**	**E**	**M**	**E**	**M**
Chen *et al*. [7]	2011	RC	68	59	48	29	128	170	4.3(3 to 12)	7.3(5 to 22)
D'Haens *et al*. [8]	2009	RC	60	60	38	30	NA	NA	NA	NA
Higgins *et al*. [9]	2008	RC	16	25	14	20	117	152	3	5.3
O'Maley *et al*. [10]	2008	RC	25	25	14(21)^a^	17(22)^a^	176.4	264.6	3.92 (3 to 9)	4.84 (3 to 9)
Choe *et al*. [11]	2008	RC	12	11	10	8	NA	NA	NA	NA
Casler *et al*. [12]	2005	RC	15	15	10	12	255.33	245.73	4.4(2 to 7)	5.73(3 to 8)
Atkinson *et al*. [13]	2008	RC	21	21	21	21	NA	NA	3.0(1 to 10)	4.5(2 to 9)
Sheehan *et al*. [14]	1999	RC	26	44	7(16)^a^	15(36)^a^	162	204	NA	NA
White *et al.*[15]	2004	RC	50	50	NA	NA	NA	NA	3.7	5.4
Razak *et al*. [16]	2013	RC	40	40	15(16)^a^	8(14)^a^	202	169	6 ± 7.5	8 ± 6.7
Messerer *et al*. [17]	2011	RC	82	82	61	41	NA	NA	NA	NA
Cappabianca *et al*. [18]	1999	RC	10	20	9	14	NA	NA	3.1 ± 0.4	6.2 ± 0.3
Koren *et al*. [19]	1999	RC	20	20	NA	NA	NA	NA	3.7(3 to 4)	7(6 to 10)
Duz *et al*. [20]	2008	RC	28	40	15	20	NA	NA	NA	NA
Neal *et al*. [21]	2007	RC	14	15	NA	NA	NA	NA	3.4	8.3

^a^Only the cases in brackets were followed to evaluate resection rate. *E*, endoscopic group; *GTR*, gross tumor removal; *M*, microscopic group; *NA*, not available; *RC*, retrospective cohort study.

**Table 2:** Characteristics of quality grade and cases of complications for included studies

**Study**	**Quality grade**	**Cases of epistaxis**		**Cases of CSF leak**		**Cases of DI**		**Cases of meningitis**		**Cases of septal perforation**		**Cases of hypopituitarism**	
		**E**	**M**	**E**	**M**	**E**	**M**	**E**	**M**	**E**	**M**	**E**	**M**
Chen *et al*. [7]	I	1	1	3	2	2	3	0	1	1	2	0	1
D'Haens *et al*. [8]	I	1	1	6	1	NA	NA	1	0	NA	NA	1	0
Higgins *et al.*[9]	I	0	0	1	1	5	7	NA	NA	1	4	1	2
O'Maley *et al*. [10]	I	0	0	3	1	1	4	0	1	NA	NA	0	1
Choe *et al*. [11]	I	0	0	2	2	1	1	0	1	NA	NA	1	3
Casler *et al*. [12]	I	0	1	4	3	3	2	NA	NA	0	3	NA	NA
Atkinson *et al*. [13]	I	0	0	3	2	3	3	NA	NA	NA	NA	NA	NA
Sheehan *et al*. [14]	I	0	0	3	7	1	0	NA	NA	0	2	NA	NA
White *et al*. [15]	I	1	8	6	7	11	11	1	0	0	1	NA	NA
Razak *et al*. [16]	I	NA	NA	4	6	4	11	1	0	NA	NA	NA	NA
Messerer *et al*. [17]	I	4	1	10	7	7	8	3	4	NA	NA	5	9
Cappabianca *et al*. [18]	II	0	0	0	0	4	2	NA	NA	NA	NA	NA	NA
Koren *et al*. [19]	II	0	2	4	5	NA	NA	NA	NA	2	6	NA	NA
Duz *et al*. [20]	II	0	0	8	10	NA	NA	NA	NA	NA	NA	NA	NA
Neal *et al.*[21]	II	0	0	4	8	1	5	NA	NA	NA	NA	NA	NA

*CSF*, cerebrospinal fluid; *DI*, diabetes insipidus; *NA*, not available.

Reviewing the characteristics of the surgical procedures of the included studies, O’Maley *et al*. [10] reported 25 cases separately in each surgery group; however, only 21 cases in the endoscopic group and 22 cases in the microscopic group were followed to evaluate the resection rate. Sixteen cases in the endoscopic group and 36 cases in the microscopic group were followed to evaluate the resection rate in Sheehan’s study [14], and 16 cases in the endoscopic group and 14 cases in the microscopic group were followed to evaluate the resection rate in Razak’s study [16]. The hospital stay and the operative time were not available for the meta-analysis because standard deviations either were not reported by, or could not be computed for, most of the reports.

We used meta-analytical techniques to obtain pooled estimates rates of post-operative outcomes and complications. Reviewing the characteristics of the surgical procedures, twelve studies [7-14,16-18,20] (endoscopic group = 365, microscopic group = 405) reported data on GTR. A fixed effects model was used because there was no evidence of significant heterogeneity (*X*^2^ = 12.11, *P* = 0.28, I^2^ = 17.4%). The proportion of patients with GTR was significantly different between the endoscopic group and the microscopic group (OR = 1.86, 95% CI 1.36 to 2.54) (Figure 2.1). A higher rate of GTR was performed in the endoscopic group than in the microscopic group (71.8% versus 58.0%). A sensitivity analysis was performed by removing two studies [18,20], and the outcome of the analysis revealed a significant difference between the endoscopic group and the microscopic group, which was consistent with previous results (OR = 1.93, 95% CI 1.38 to 2.70) (Figure 3.1). The Begg’s Test (*P* = 0.586) and Egger’s Test (*P* = 0.590) showed no publication bias.

**Figure 2:**
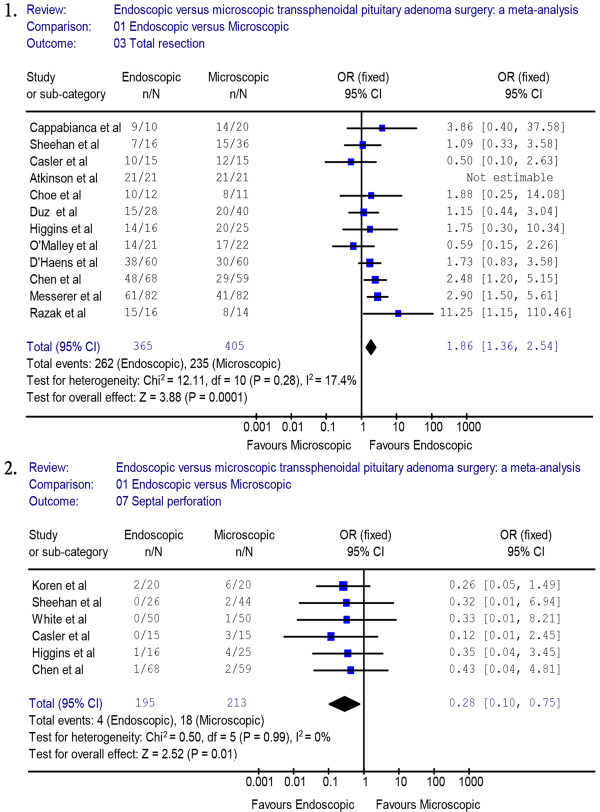
**Forest plot of the odd ratios and 95% CI for GTR and septal perforation in patients who had endoscopic and microscopic pituitary adenoma surgery.** CI, confidence interval; GTR, gross tumor removal.

**Figure 3:**
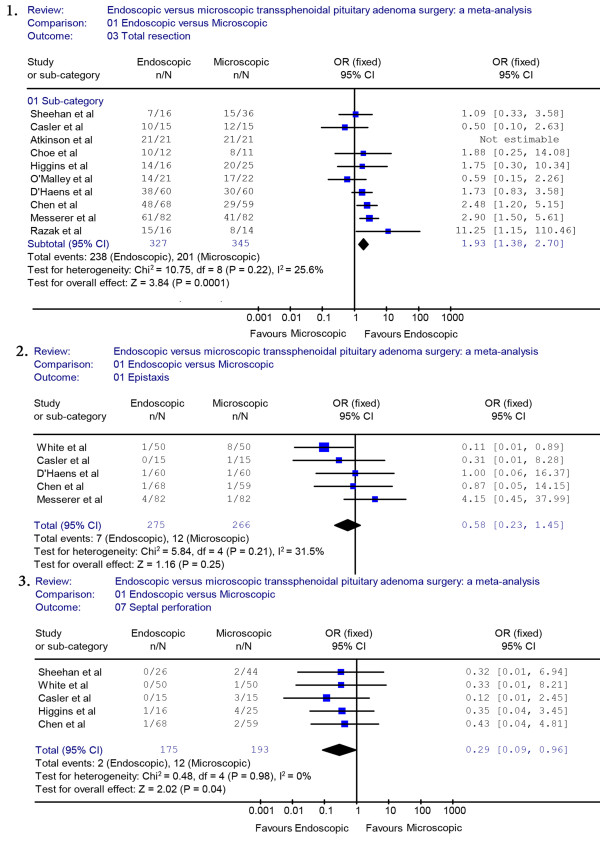
**Sensitivity analyses of GTR, epistaxis and septal perforation.** GTR, gross tumor removal.

Six studies reported on post-operative septal perforation. The difference between the endoscopic and the microscopic groups was statistically significant (OR = 0.28, 95% CI 0.10 to 0.75) (Figure 2.2). The pooled estimate of the overall proportions for the endoscopic and microscopic groups was 2.1% versus 8.5%, respectively. The proportion of septal perforation was significantly lower in those who had endoscopic surgery. A sensitivity analysis was performed by removing one study [19], and the outcome of the analysis revealed a significant difference between the endoscopic and microscopic groups, which was consistent with previous results (OR = 0.29, 95% CI 0.09 to 0.96) (Figure 3.3).

Thirteen studies (endoscopic group = 477, microscopic group = 507) reported data on CSF leak. A fixed effects model was used because there was no evidence of significant heterogeneity (*X*^2^ = 7.88, *P* = 0.85, I^2^ = 0%). The occurrence rate of CSF leak was not significantly different between the endoscopic group and the microscopic group (OR = 1.11, 95% CI 0.75 to 1.63) (Figure 4.1). The incidence rate of CSF leak in the endoscopic group was not significantly lower than in the microscopic group (12.8% versus 12.2%, respectively). A sensitivity analysis of the CSF leak was performed by removing three studies [19-21], and the outcome of the analysis did not reveal a significant difference between the endoscopic and the microscopic groups, which was consistent with previous results (OR = 1.26, 95% CI 0.8 to 2.0) (Figure 5.1). The Begg’s Test (*P* = 0.2) and Egger’s Test (*P* = 0.28) for CSF leak showed no publication bias.

**Figure 4:**
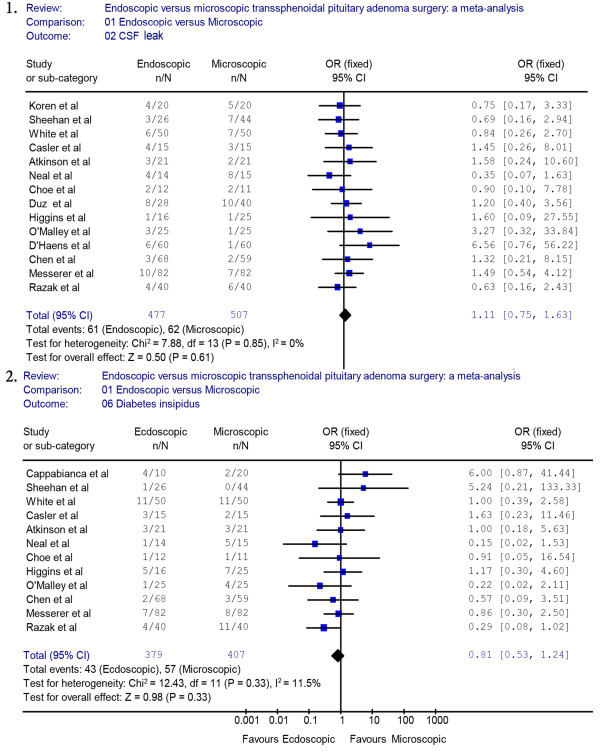
**Forest plot of the odd ratios and 95% CI for CSF leak, DI who had endoscopic and microscopic pituitary adenoma surgery.** CI, confidence interval; CSF, cerebrospinal fluid; DI, diabetes insipidus.

**Figure 5:**
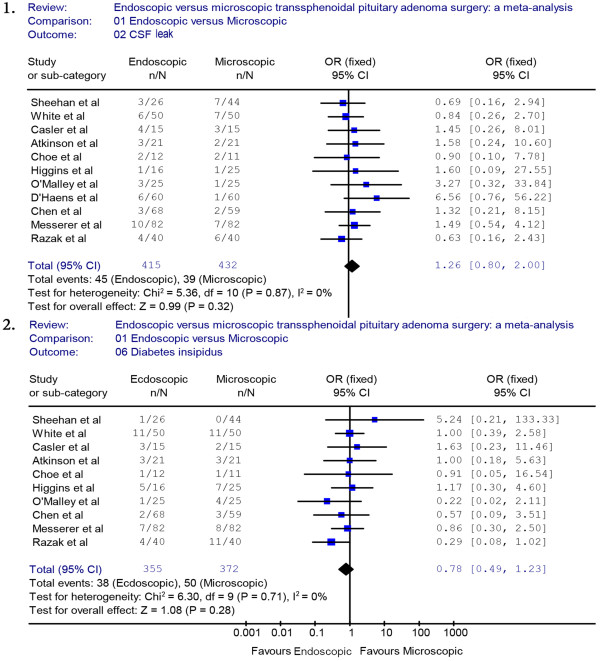
**Sensitivity analyses of CSF leak and DI.** CSF, cerebrospinal fluid; DI, diabetes insipidus.

A fixed effects model was used for DI because there was no evidence of significant heterogeneity (*X*^2^ = 12.43, *P* = 0.33, I^2^ = 11.5%). The occurrence rate of DI was 11.3% in the endoscopic group and 14.0% in the microscopic group. The pooled estimates of the overall proportions showed no significant difference between the endoscopic and microscopic groups based on the results of 11 studies (OR = 0.81, 95% CI 0.53 to 1.24) (Figure 4.2). A sensitivity analysis was performed by removing two studies [18,21], and the outcome of the analysis did not reveal a significant difference between the endoscopic and the microscopic groups, which was consistent with previous results (OR = 0.78, 95% CI 0.49 to 1.23) (Figure 5.2). The Begg’s Test (*P* = 0.89) and Egger’s Test (*P* = 0.81) for DI indicated that there was no publication bias.

Six studies reported data on pituitary hypopituitarism. A fixed effects model was used because there was no evidence of significant heterogeneity (*X*^2^ = 1.85, *P* = 0.87, I^2^ = 0%). In those studies, we found that the proportion of pituitary dysfunction was 3% in the endoscopic group and 6.1% in the microscopic group. The endoscopic approach appeared to reduce the occurrence rate of hypopituitarism. However, the pooled estimates of the meta-analysis showed that there was no significant difference between the two groups for the rate of complications (OR = 0.53, 95% CI 0.23 to 1.20) (Figure 6.1).

**Figure 6:**
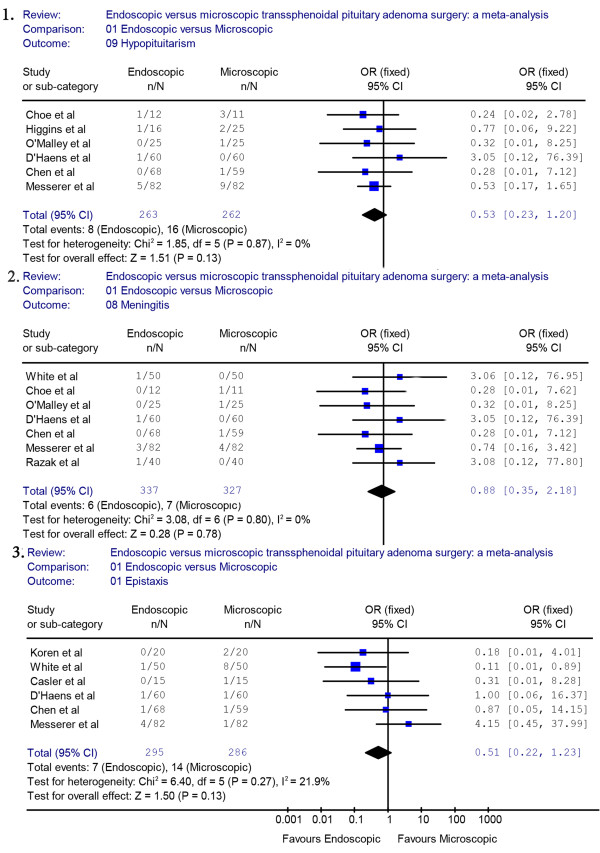
**Forest plot of the odd ratios and 95% CI for hypopituitarism, meningitis and epistaxis in patients who had endoscopic and microscopic pituitary adenoma surgery.** CI, confidence interval.

Seven studies reported data on post-operative meningitis. A fixed effects model was used because there was no evidence of significant heterogeneity (*X*^2^ = 3.08, *P* = 0.80, I^2^ = 0%). The pooled complication OR for meningitis indicated that the occurrence rate of meningitis was not significantly different between the endoscopic and the microscopic groups (OR = 0.88, 95% CI 0.35 to 2.18) (Figure 6.2).

The proportion of patients with post-operative epistaxis was not significantly different between the endoscopic group and the microscopic group (OR = 0.51, 95% CI 0.22 to 1.23) (Figure 6.3). The proportion of epistaxis was not significantly different between the endoscopic and the microscopic groups (2.4% versus 4.9%). A sensitivity analysis was performed by removing one study [19], and the outcome of the analysis revealed no difference between the endoscopic and microscopic groups, which was consistent with previous results (OR = 0.58, 95% CI 0.23 to 1.45) (Figure 3.2).

A total of six endoscopic studies reported data on the length of operation with a mean time of 173 ± 5 minutes versus 201 ± 46 minutes for the microscopic groups (Table 3). The difference was not statistically significant (*P* = 0.259). However, 10 endoscopic studies reported data on the hospital stay that showed a mean time of 3.8 ± 0.9 days in the endoscopic group while 6.3 ± 1.3 days in the microscopic group, respectively. The difference between the two approaches was statistically significant (*P* = 0.0002), and the hospital stay was significantly shorter in the endoscopic group than in the microscopic group.

**Table 3:** Comparison of the length of operation and hospital stay between the endoscopic and microscopic approaches

	**Endoscopic group**	**Microscopic group**	***P *** **Value**^**a**^
**Length of operation**			
Mean time, min	173 ± 5 minutes	201 ± 46 minutes	0.349
Total number of studies	6^b^(190)	6^b^(208)	
**Length of hospital stay**			
Mean time, days	3.8 ± 0.9 days	6.3 ± 1.3 days	P = 0.00017
Total numberof studies	10^c^(239)	10^c^(250)	

^a^Statistical analysis performed using equal-variance *t*-test; ^b^References [7,9-11,14,16]; ^c^References [7,9,10,12,13,15,16,18],[19,21].

## Discussion

Over the last century, pituitary adenoma surgery has evolved from a craniotomy approach toward less invasive approaches. In the past twenty years, there is growing evidence to support the use of endoscopic techniques as an alternative approach in the treatment of pituitary adenomas [16,22-26]. Endoscopy can expand the limits of the surgeons’ performance of transsphenoidal surgery, improving visualization and removing tumors that they could not access before. It is likely that its characteristic of minimal invasiveness explains the positive outcomes and lower proportion of post-operative complications of endoscopic procedures in comparison with the microscopic approach.

Several authors have discussed the potential outcomes of the endoscopic technique. DeKlotz *et al*. [22] used a meta-analysis to reveal the superior rate of GTR (79% versus 65%, *P* < 0.0001) as well as the lower rates of CSF leak (5% versus 7%, *P* < 0.01), septal perforation (0% versus 5%) and post-operative epistaxis (1% versus 4%, *P* < 0.0001) for the endoscopic approach compared with the sublabial approach. Rotenberg *et al*. [23] concluded that the two approaches had similar outcomes (GTR, hormonal abnormality resolution) but that the endoscopic approach was associated with fewer complications as well as a shorter hospital stay and length of operation. Goudakos *et al*. [24] demonstrated that the rates of GTR/CSF leakage were similar between the two techniques. However, the study also revealed a lower incidence of post-operative DI and a shorter hospital stay in the studied endoscopic groups. Other systematic reviews also support the safety and short-term efficacy of endoscopic pituitary surgery [16,25]. Interestingly, Ammirati *et al*. [27] recently reported a meta-analysis concluding that endoscopic removal of pituitary adenoma, in the short term, does not seem to confer any advantages over the microscopic technique and the incidence of vascular complications was higher with endoscopic than with microscopic removal of pituitary adenomas.

How can the reported difference be explained? The primary explanation is that most of the previous reports pertain to single-armed studies in the absence of a reliable comparison. Second, the inclusion and exclusion criteria are key factors in each study, which may lead to different conclusions. In addition, the complication rate in microscope-based surgery is already low and the rates of GTR are high. Demonstrating a statistically significant difference between endoscopic and microscopic techniques will require a larger number of cases. Furthermore, a learning curve [28,29] is anticipated because the endoscopic approach is a newer technique, and gradual improvement in outcomes will occur as the cumulative experience increases over time. Future studies are required to resolve the learning curve issues.

However, Doglietto *et al*. [30] reported that it may not be the time to conduct meta-analyses of endoscopic skull base surgery but it is certainly an appropriate time to collected data prospectively. As we know, the development of a new surgical technique often begets criticism due to the possibility of a learning curve. To date, no large, prospective, randomized study has been performed to compare outcomes between endoscopic and microsurgical transsphenoidal techniques. We believe that the endoscopic technique, after a first phase of un-acceptance, will prove its efficacy or superiority to ‘classic’ techniques and a systematic retrospective study of published results to compare microsurgical and endoscopic techniques in the treatment of pituitary adenomas may provide important significant guidance for further research.

The results of our meta-analysis clearly favor the endoscopic approach for pituitary surgery over the microscopic approach. The endoscopic approach yielded a significantly improved rate of GTR with lower rate of post-operative septal perforation and a shorter length of hospital stay. There were no significant differences between the two approaches for meningitis, epistaxis, DI, CSF leak, hypopituitarism and the overall length of operation time. It is important to recognize that the above analysis represents only the results of early outcomes and complications. There are few published long-term studies following these patients beyond the initial post-operative period.

The endoscopic technique appears to provide a higher rate of GTR compared with microscopic resection. The results from our analysis showed that the rate of GTR was significantly higher in the endoscopic group than in the microscopic group (71.8% versus 58.0%). Unfortunately, a subgroup analysis of the GTR based on the size of the pituitary adenoma was not feasible in our analysis because of the lack of available data. The actual size of the tumors was not recorded in most reports. When size was reported, it was infrequently correlated to actual surgical outcomes.

The primary complication for the majority of patients undergoing pituitary surgery is CSF leak. The currently accepted view is that the success of reconstructive techniques following dissection should be a major determinant of post-operative CSF leak [18]. Endoscopy appears to have a huge advantage in reconstruction because it improves visualization. However, in our study, the rate of post-operative leaks was similar (12.8% versus 12.2% for the endoscopic and microscopic groups, respectively). The main reason for this similarity may be that the improved exposure during endoscopic surgery would encourage the surgeons to extend the limits of their operation more aggressively, which may offset the minimally invasive nature of endoscopic resections and increase the rate of post-operative CSF leak.

It is important to note that there are some potential limitations to this study. First, only English-language articles were considered, which means that some relevant studies in other languages may have been omitted from our meta-analysis; this may have introduced a language bias. In addition, all of the studies in our analysis are retrospective studies, which are associated with several methodological issues including selection bias, incomplete data, and a lack of standardization in the study intervention. Unfortunately, large randomized prospective studies comparing the two techniques are not available at present. Moreover, publications to date represent the results of short-term outcomes and complications. There are few published long-term studies that follow these patients beyond the initial post-operative period. Therefore, we expect our conclusions to be interpreted with caution.

## Conclusions

In conclusion, the results of our meta-analysis support the safety and short-term effectiveness of endoscopic transsphenoidal pituitary adenoma surgery. The endoscopic approach is associated with a higher rate of GTR, decreased hospital stay and reduced observed post-operative complication (septal perforation). Future studies with a long-term follow-up are required to determine the outcomes and complications of endoscopic pituitary surgery.

## Abbreviations

CI: confidence interval; CSF: cerebrospinal fluid; DI: diabetes insipidus; GTR: gross tumor removal; NA: not available; OR: odds ratio; RC: retrospective cohort study.

## Competing interests

The authors declare that they have no competing interests.

## Authors’ contributions

CZ and YW designed the study; YG drafted the manuscript; SX and YG searched the papers and extracted the data; YG and CD participated in the statistical analysis; YZ, YW, QL and JJ assisted in the critical appraisal of the included studies. All authors read and approved the final manuscript.

## References

[B1] Horsley V Remarks on ten consecutive cases of operations upon the brain and cranial cavity to illustrate the details and safety of the method employed Br Med J 1887 1 863 865 10.1136/bmj.1.1373.863 20751874 PMC2534542

[B2] Schloffer H Erfolgreiche operation eines hypophysentumors auf nasalem wege Wien Klin Wochenschr 1907 20 621 624

[B3] Cushing H Intracranial Tumors: Notes Upon a Series of Two-Thousand Verified Cases With Surgical-Mortality Percentages Pertaining Thereto 1932 Springfield, IL: Charles C Thomas 69 79

[B4] Hardy J Transsphenoidal removal of pituitary adenomas Union Med Can 1962 91 933 945 13952789

[B5] Jankowski R Auque J Simon C Marchal JC Hepner H Wayoff M Endoscopic pituitary tumor surgery Laryngoscope 1992 102 198 202 1738293 10.1288/00005537-199202000-00016

[B6] Wells GA Shea B O’Connell D Peterson J Welch V Losos M Tugwell P The Newcastle-Ottawa Scale (NOS) for assessing the quality if nonrandomized studies in meta-analyses 2008 Available at: http://www.ohri.ca/programs/clinical_epidemiology/oxford.htm

[B7] Cheng RX Tian HL Gao WW Li ZQ A comparison between endoscopic transsphenoidal surgery and traditional transsphenoidal microsurgery for functioning pituitary adenomas J Int Med Res 2011 39 1985 1993 10.1177/147323001103900545 22118003

[B8] D’Haens J Van Rompaey K Stadnik T Haentjens P Poppe K Velkeniers B Fully endoscopic transsphenoidal surgery for functioning pituitary adenomas: a retrospective comparison with traditional transsphenoidal microsurgery in the same institution Surg Neurol 2009 72 336 340 10.1016/j.surneu.2009.04.012 19604551

[B9] Higgins TS Courtemanche C Karakla D Strasnick B Singh RV Koen JL Han JK Analysis of transnasal endoscopic versus transseptal microscopic approach for excision of pituitary tumors Am J Rhinol 2008 22 649 652 19178807 10.2500/ajr.2008.22.3246

[B10] O'Malley BW Jr Grady MS Gabel BC Cohen MA Heuer GG Pisapia J Bohman LE Leibowitz JM Comparison of endoscopic and microscopic removal of pituitary adenomas: single-surgeon experience and the learning curve Neurosurg Focus 2008 25 E10 19035697 10.3171/FOC.2008.25.12.E10

[B11] Choe JH Lee KS Jeun SS Cho JH Hong YK Endocrine outcome of endoscopic endonasaltranssphenoidal surgery in functioning pituitary adenomas J Korean Neurosurg Soc 2008 44 151 155 10.3340/jkns.2008.44.3.151 19096666 PMC2588303

[B12] Casler JD Doolittle AM Mair EA Endoscopic surgery of the anterior skull base Laryngoscope 2005 115 16 24 10.1097/01.mlg.0000150681.68355.85 15630358

[B13] Atkinson JL Young WF Jr Meyer FB Davis DH Nippoldt TB Erickson D Vella A Natt N Abboud CF Carpenter PC Sublabialtransseptal vs transnasal combined endoscopic microsurgery in patients with Cushing disease and MRI-depicted microadenomas Mayo Clin Proc 2008 83 550 553 18452684 10.4065/83.5.550

[B14] Sheehan MT Atkinson JL Kasperbauer JL Erickson BJ Nippoldt TB Preliminary comparison of the endoscopic transnasal vs the sublabialtransseptal approach for clinically nonfunctioning pituitary macroadenomas Mayo Clin Proc 1999 74 661 670 10.4065/74.7.661 10405694

[B15] White DR Sonnenburg RE Ewend MG Senior BA Safety of minimally invasive pituitary surgery (MIPS) compared with a traditional approach Laryngoscope 2004 114 1945 1948 10.1097/01.mlg.0000147925.04605.cc 15510019

[B16] Razak AA Horridge M Connolly DJ Warren DJ Mirza S Muraleedharan V Sinha S Comparison of endoscopic and microscopic transsphenoidal pituitary surgery: early results in a single centre Br J Neurosurg 2013 27 40 43 10.3109/02688697.2012.703353 22834971

[B17] Messerer M De Battista JC Raverot G Kassis S Dubourg J Lapras V Trouillas J Perrin G Jouanneau E Evidence of improved surgical outcome following endoscopy for nonfunctioning pituitary adenoma removal Neurosurg Focus 2011 30 E11 21456922 10.3171/2011.1.FOCUS10308

[B18] Cappabianca P Alfieri A Colao A Ferone D Lombardi G de Divitiis E Endoscopic endonasaltranssphenoidal approach: an additional reason in support of surgery in the management of pituitary lesions Skull Base Surg 1999 9 109 117 10.1055/s-2008-1058157 17171126 PMC1656809

[B19] Koren I Hadar T Rappaport ZH Yaniv E Endoscopic transnasaltranssphenoidal microsurgery versus the sublabial approach for the treatment of pituitary tumors: endonasal complications Laryngoscope 1999 109 1838 1840 10.1097/00005537-199911000-00022 10569418

[B20] Duz B Harman F Secer HI Bolu E Gonul E Transsphenoidal approaches to the pituitary: a progression in experience in a single centre Acta Neurochir 2008 150 1133 1138 10.1007/s00701-008-0135-y 18958390

[B21] Neal JG Patel SJ Kulbersh JS Osguthorpe JD Schlosser RJ Comparison of techniques for transsphenoidal pituitary surgery Am J Rhinol 2007 21 203 206 10.2500/ajr.2007.21.2981 17424881

[B22] DeKlotz TR Chia SH Lu W Makambi KH Aulisi E Deeb Z Meta-analysis of endoscopic versus sublabial pituitary surgery Laryngoscope 2012 122 511 518 10.1002/lary.22479 22252670

[B23] Rotenberg B Tam S Ryu WH Duggal N Microscopic versus endoscopic pituitary surgery: a systematic review Laryngoscope 2010 120 1292 1297 10.1002/lary.20949 20578228

[B24] Goudakos JK Markou KD Georgalas C Endoscopic versus microscopic transsphenoidal pituitary surgery: a systematic review and meta-analysis Clin Otolaryngol 2011 36 212 220 10.1111/j.1749-4486.2011.02331.x 21752205

[B25] Tabaee A Anand VK Barron Y Hiltzik DH Brown SM Kacker A Mazumdar M Schwartz TH Endoscopic pituitary surgery: a systematic review and meta-analysis J Neurosurg 2009 111 545 554 10.3171/2007.12.17635 19199461

[B26] Shah S Har-El G Diabetes insipidus after pituitary surgery:incidence after traditional versus endoscopic transsphenoidal approaches Am J Rhinol 2001 15 377 379 11777244

[B27] Ammirati M Wei L Ciric I Short-term outcome of endoscopic versus microscopic pituitary adenoma surgery: a systematic review and meta-analysis J Neurol Neurosurg Psychiatry 2013 84 843 849 10.1136/jnnp-2012-303194 23243265 PMC3717601

[B28] Koc K Anik I Ozdamar D Cabuk B Keskin G Ceylan S The learning curve in endoscopic pituitary surgery and our experience Neurosurg Rev 2006 29 298 305 10.1007/s10143-006-0033-9 16937143

[B29] Leach P Abou-Zeid AH Kearney T Davis J Trainer PJ Gnanalingham KK Endoscopic transsphenoidal pituitary surgery: evidence of an operative learning curve Neurosurgery 2010 67 1205 1212 10.1227/NEU.0b013e3181ef25c5 20871457

[B30] Doglietto F Maira G Endoscopic skull base surgery: probably not the time for meta-analyses but certainly for prospectively collected data World Neurosurg 2013 80 784 786 10.1016/j.wneu.2013.07.097 23924969

